# Uranium in the Surrounding of San Marcos-Sacramento River Environment (Chihuahua, Mexico)

**DOI:** 10.1100/2012/616430

**Published:** 2012-03-12

**Authors:** Marusia Rentería-Villalobos, Manuel Reyes Cortés, Juan Mantero, Guillermo Manjón, Rafael García-Tenorio, Eduardo Herrera, Maria Elena Montero-Cabrera

**Affiliations:** ^1^Advanced Material Research Center, CIMAV, Miguel de Cervantes 120, 31109 Chihuahua, CHIH, Mexico; ^2^Facultad de Ingenieria, Universidad Autonoma de Chihuahua, Nuevo Campus, 31000 Chihuahua, CHIH, Mexico; ^3^Applied Nuclear Physics Group, Departamento de Física Aplicada, ETS Arquitectura, University of Seville, Avenida Reina Mercedes s/n, 41012 Seville, Spain

## Abstract

The main interest of this study is to assess whether uranium deposits located in the San Marcos outcrops (NW of Chihuahua City, Mexico) could be considered as a source of U-isotopes in its surrounding environment. Uranium activity concentrations were determined in biota, ground, and surface water by either alpha or liquid scintillation spectrometries. Major ions were analyzed by ICP-OES in surface water and its suspended matter. For determining uranium activity in biota, samples were divided in parts. The results have shown a possible lixiviation and infiltration of uranium from geological substrate into the ground and surface water, and consequently, a transfer to biota. Calculated annual effective doses by ingestion suggest that U-isotopes in biota could not negligibly contribute to the neighboring population dose. By all these considerations, it is concluded that in this zone there is natural enhancement of uranium in all environmental samples analyzed in the present work.

## 1. Introduction

The State of Chihuahua is located at the north of Mexico. Its capital is Chihuahua City, which is located in a semiarid region that uses, above all else, San Marcos-Sacramento aquifer waters for human consumption. 

In Chihuahua State, about 30 uranium anomalies have been found; most of them are located near Chihuahua City [1]. Although the uranium deposit of Peña Blanca has been studied in previous works by other authors, there are at least two other zones in the area that could contribute with radioactive minerals to the Chihuahua City basin: the Pastorias zone at SW and the San Marcos zone at NW from Chihuahua City. This later area was studied in this paper regarding the pollution caused by natural occurring uranium. 

To assess the radiological contamination from either natural or anthropogenic sources, it is important to understand the behavior of radionuclides released to the environment [2]. The radioisotopes ^238^U, ^235^U, and ^232^Th are the first radionuclides of three natural decay chains, respectively. 

Uranium is occurring naturally in the earth crust, and its average content will vary as a function of rock type. Generally uranium is concentrated in igneous, metamorphic, and sedimentary rocks: granite, lignite, and phosphate deposits [3]. The uranium geological cycles begin by weathering processes in the earth's crust and continue with mobilization by surface and ground water. In rock systems, secular radioactive equilibrium is common, while surface and ground waters are characterized by significant disequilibria. Uranium exists dominantly in the +4 and +6 oxidation states in most geologic environments [4, 5]. The uranium transport generally occurs in oxic natural waters as uranyl species (U^+6^), mostly complexed with hydroxide, carbonate, fluoride, sulphate, or phosphate [6]. Natural uranium concentrations in ground water ranges from 0.1 to 10 mg L^−1^ (or ppm) [7, 8], while dissolved uranium concentration in rivers ranges from 0.01 to 100 *μ*g kg^−1^ (or ppb) and have a global average of about 0.3 ppb [4]. 

Likewise, the uranium isotopes in soil and water may be transferred to the plants and animals. The main sources of radionuclides uptaken by plants are the geologic substratum, the atmospheric fallout, or direct discharge from industries into the surrounding environment. It is necessary to evaluate the soil to plant transfer of uranium isotopes, because it is the beginning of the radioactive contamination in the food-chain. 

Liquid scintillation analysis (LSA) is a useful tool in the analysis of environmental level radionuclide concentration. This technique has been widely used and recognized for its validity by many authors [9–22]. The liquid scintillation counting (LSC) provides discrimination between alpha and beta radiations, due to difference behavior of their pulse decay. LSA combines chemical separation by liquid-liquid extraction with the measurement of alpha activity by liquid scintillation. The main advantages of the method are the easy sample preparation and the high counting efficiency (about 100%). 

The aim of this study was to assess the transport of uranium from minerals outcrops located in San Marcos area (NW of Chihuahua City, Mexico) into the close environment, by means of the naturally occurring radioactivity levels in surface and ground water, plants, and fish.

## 2. Material and Methods

### 2.1. Study Area

**Figure 1:**
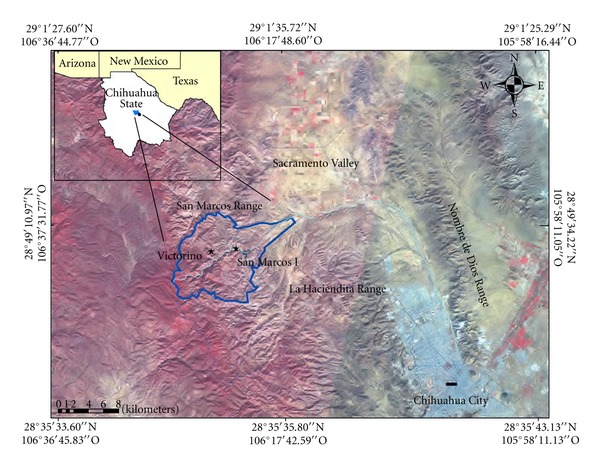
Location of the study area in Chihuahua, Mexico.

In Figure 1 is showed the study area. The study was carried out in the San Marcos-Sacramento area located at northwest of Chihuahua city, Mexico. San Marcos area is a rhyolitic volcanic system, showing mainly rhyolitic tuffs and some Upper Cenozoic intermediate volcanic sequences [23]. This range formation is a so-called “caldera”, which has uranium minerals deposits. Its uranium mineralogical characterization showed the following radioactive species: uranophane, metatyuyamunite, uraninite, becquerelite, and masuyite [24]. San Marcos range formation includes San Marcos River, San Marcos dam, and at least two uranium outcrops. The uranium mineral outcrops are Victorino and San Marcos I, that are of hydrothermal origin [23]. 

The Sacramento valley is surrounding by 3 mountain ranges: San Marcos (West), La Haciendita (South), and Nombre de Dios (East). In this valley are located the agricultural wells from which the water was extracted for this study. 

The San Marcos-Sacramento area has a semiarid climate where low weathering prevails, so the study of uranium transport can reveal its impact to the closer ecosystem [25]. The San Marcos River is a typical arid river where the water runs only during the rainy season (July–September). The river passes close to the outcrops. The river water crosses firstly Victorino mineral outcrop and next the San Marcos I mineral outcrop, thereafter the river reaches the dam where most water is stored. The river and the dam represent the main water-supply to agricultural areas in that region.

### 2.2. Sampling

The samples analyzed in this work were collected in two different sampling campaigns, as is explained below. Figure 1 shows the sampling location of all kinds of samples analyzed in this paper.

#### 2.2.1. First Sampling (2005, Rainy Reason)

This sampling was carried out on the rainy period July-August of 2005. Agricultural wells from Sacramento valley and closer to San Marcos outcrops were sampled to determine natural radioactivity concentrations in groundwater. Two groundwater samples were taken, wells 1 and 2. *Baccharis salicifolia* (commonly called “jarilla”), which is the typical wild plant species that grows up to riverside in this region, was selected to determine uranium concentration in plants. This plant species is not involved directly in the human food chain, but the information about concentration levels and its translocation of uranium will contribute to understand the transfer mechanisms to plants that belong to the human diet. In this sampling, only one sample of that plant was taken. Additionally, sixteen fish samples of *Cichlasoma labridens* (commonly called “mojarra”) were collected from the dam. This fish type is consumed by some people who go fishing to this place. The determination of uranium activity concentration was made on the clean muscles from fish.

#### 2.2.2. Second Sampling (2007, Dry Season)

Additionally to the 2005 sampling, other 15 groundwater samples (wells from 3 to 17) were collected in the dry period from October 2006 to January 2007. The groundwater samples were taken from agricultural wells present within Sacramento valley. Surface water sampling was also carried out on both river and dam. Surface water samples were collected in the still dry period January–March 2007. Due to the semiarid climate and under the dry conditions, the water frequently flows by subsurface way, having as a consequence high residence time in contact with the bedrock; water only emerges at some points. The San Marcos River was sampled in several points along its path. In this campaign, six water samples were taken from different points at the dam. In order to improve the knowledge on the uranium concentration into plants, five plant samples were collected at the same points and at same time as surface water (January–March of 2007). 

The ground and surface water samples (on both sampling campaigns) were collected in polyethylene 5 L containers. Geographic coordinates, temperature, total dissolved solids (TDS), and pH parameters were measured in situ. The water samples were filtered (20–25 *μ*m pore diameter) to remove suspended matter. Here we will call “uranium dissolved” to uranium concentration in filtered water: water that passed through 20–25 *μ*m filter.

### 2.3. Experimental

U-isotopes concentration was determined by two different techniques: high-resolution alpha spectrometry (HRAS) and liquid scintillation counting (LSC).

#### 2.3.1. High-Resolution Alpha Spectrometry

Uranium in samples collected in 2005 (two groundwater samples, one plant sample, and 16 fish samples) was determined using HRAS at the Applied Nuclear Physics Group Laboratory of the University of Seville, Spain. 

The groundwater samples were filtered and acidified with nitric acid to pH 2. The plant sample was divided in root, stem, and leaves. Once separated, subsamples were washed with distilled water to remove any trace of sediment or soil particles and then were dried. In fish only the muscles was analyzed. 

All samples were spiked with ^232^U and put under the radioanalytical analysis procedure. Total sample dissolution was performed by atmospheric acid digestion using 8 M HNO_3_ and H_2_O_2_. TBP was used as a uranium extracting agent in these samples [26–28]. Then, uranium was electrodeposited on stainless steel planchets [29]. An alpha-spectrometry chain Alpha Analyst (CANBERRA) was used for alpha activity measurements. Radiochemical yield was determined by the ^232^U counting rate.

#### 2.3.2. Liquid Scintillation Counting

U-isotopes concentration in samples collected in the 2006-2007 sampling campaign (15 groundwater samples, 10 surface water samples from the river, 6 surface water samples from the dam, and 5 plant samples) was determined using LSC at the Laboratory of Environmental Radiological Surveillance (LVRA) in the Advanced Materials Research Center (CIMAV), Chihuahua, Mexico. 

The surface and ground water samples were filtered to remove suspended matter. Filtered water was acidified with nitric acid to pH 2. The filtrate, suspended matter from surface water, were dried and digested with HCl and HNO_3_ solutions. Suspended matter samples were analyzed by ICP-OES for concentrations of major ions. Major ions were also analyzed in filtered water samples. In water, one duplicate for each five samples was taken as repeatability control. 

In plants, suspended matter, and surface water, uranium was isolated from the sample by solvent extraction with bis(2-ethylhexyl) phosphoric acid (HDEHP) [19]. The activity concentration of total uranium from these samples was determined using a TRIATHLER OY spectrometer. In order to test the reliability of this technique, an intercomparison exercise of different radiometric techniques for uranium determination in groundwater was organized: alpha spectrometry with semiconductor detectors; gross alpha-counting and direct evaporation; gross alpha-counting and coprecipitation; gross alpha-counting and U-extraction after coprecipitation; liquid scintillation counting and sequential extraction; portable liquid scintillation counting and cocktail extraction (LSCCE, our technique). The exercise was performed on the analysis of three groundwater samples, extracted from three different wells from Chihuahua City. The IAEA Analytical Quality Control Services procedure was used to evaluate the accuracy and precision. Most of techniques, including our LSCCE, showed a good agreement in terms of precision (95% confidence). 

In groundwater samples, uranium was extracted with URAEX extracting cocktail [30]. ^238^U- and ^234^U-specific activities were determined by PERALS (photon/electron-rejecting alpha liquid scintillation) spectrometer [31]. This technique was tested using a certified reference material (solution from High Purity Standards no. 100064), where the sample was spiked with ^232^U (SRM 4324B from NIST) for its quantification. Results for ^238^U and ^234^U were 0.601 Bq and 0.571 Bq, respectively, with relative uncertainty of 3%, while those reported from the reference material were 0.617 ± 0.002 Bq to ^238^U and 0.599 ± 0.002 Bq to ^234^U. 

## 3. Results and Discussion

### 3.1. Groundwater

**Figure 2:**
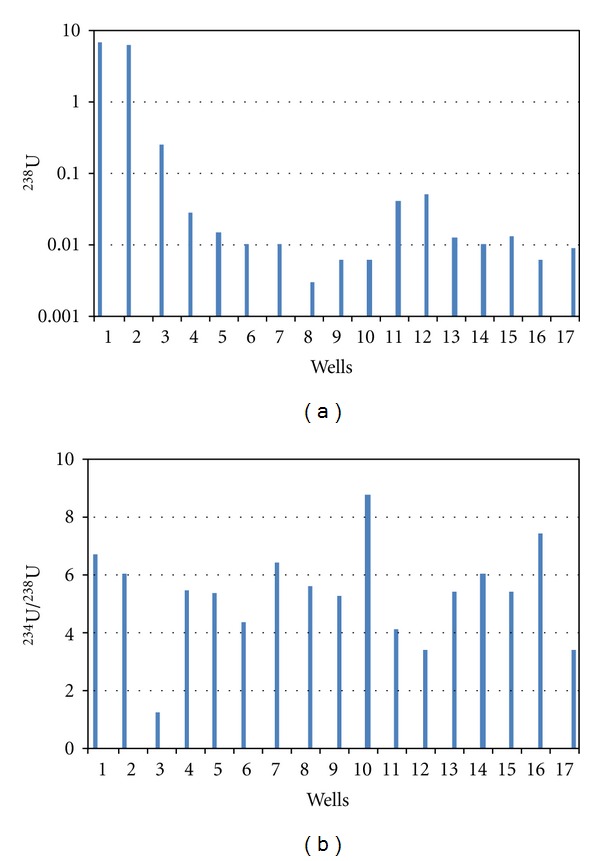
Uranium contents in underground water; (a) ^238^U activity concentration (Bq/L), (b) ^234^U/^ 238^U activity ratio. Note: water from wells 1 and 2 was sampled in 2005; water from wells 3 to 17 was sampled in 2007.

Total dissolved solids (TDS), pH, temperature (*T*) data as well as geographic coordinates of groundwater samples are shown in Table 1. In Table 2 are shown the activity concentrations of ^238^U and ^234^U isotopes, as well as their ^234^U/^238^U activity ratios (AR), for groundwater samples from wells belonging to San Marcos region. These results are also presented in Figure 2. 

In these groundwater samples, pH shows values from slightly acid to neutral, whereas TDS amounts ranged from 80 to 310 ppm. The relative uncertainty for ^238^U was below to 10% while for ^234^U was below to 4%. Analysis of pH measured in groundwater samples from wells taken in both seasons, rainy (2005) and dry (2007), did not show significant difference for a 95% confidence. However, values found for ^238^U and ^234^U activity concentrations differ significantly with 95% confidence level. 

Taking into account the recommended limit to gross alpha emitters in drinking water of 0.56 Bq/L from Mexican regulations [32], as well as the recommended limit to ^238^U contents in drinking water from the US Environmental Protection Agency of 0.37 Bq/L [33], uranium contents in water from wells 1 and 2 are exceeding by far both of them, whereas the uranium content corresponding to well 3 is just in the allowable limits. In samples from the remaining wells, uranium concentrations were below both limits. 

Most rocks in San Marcos were classified as rhyolitic in a previous work, with abundant silica; uranium contents ranged from 10 to 228 Bq/kg [34]. Uranium contents found in groundwater samples can be attributed to uranium concentrations in substrate rocks [35]. Likewise, the isotopic disequilibrium might be related directly to uranium oxidation state, which determines its solubility. Indeed, Chabaux et al. [36] and Porcelli and Swarzenski [37] have asserted that ^238^U preferably exists in its oxidation state +4 in rocks that is practically insoluble in water. However, uranium is oxidized to hexavalent state after ^238^U disintegration, which is soluble and mobile in water as uranyl ion. Also these authors have suggested that since ^234^U is resident in damaged lattice locations, it is more vulnerable to oxidation by fluids. Additionally, other authors has proposed that as uranium is released by weathering, tetravalent ^238^U is preferentially precipitated or adsorbed, while hexavalent ^234^U, oxidized during the recoil process, more readily remains in solution [37, 38]. 

In our study, a high uranium concentration in water from wells 1 and 2 was observed (see Figure 2). The main factors that are affecting that water might be lixiviation of uranium contents in rocks from San Marcos outcrops and its subsequent infiltration into subsoil. Additionally, in these groundwater samples activity ratio values are from 2 up to 6. 

Also, it was observed that the activity concentrations in groundwater dropped when the sampling site was farther from the San Marcos outcrop (Table 2). Therefore, uranium concentration in that substrate rock might be the primary source of uranium contents in water. 

**Figure 3:**
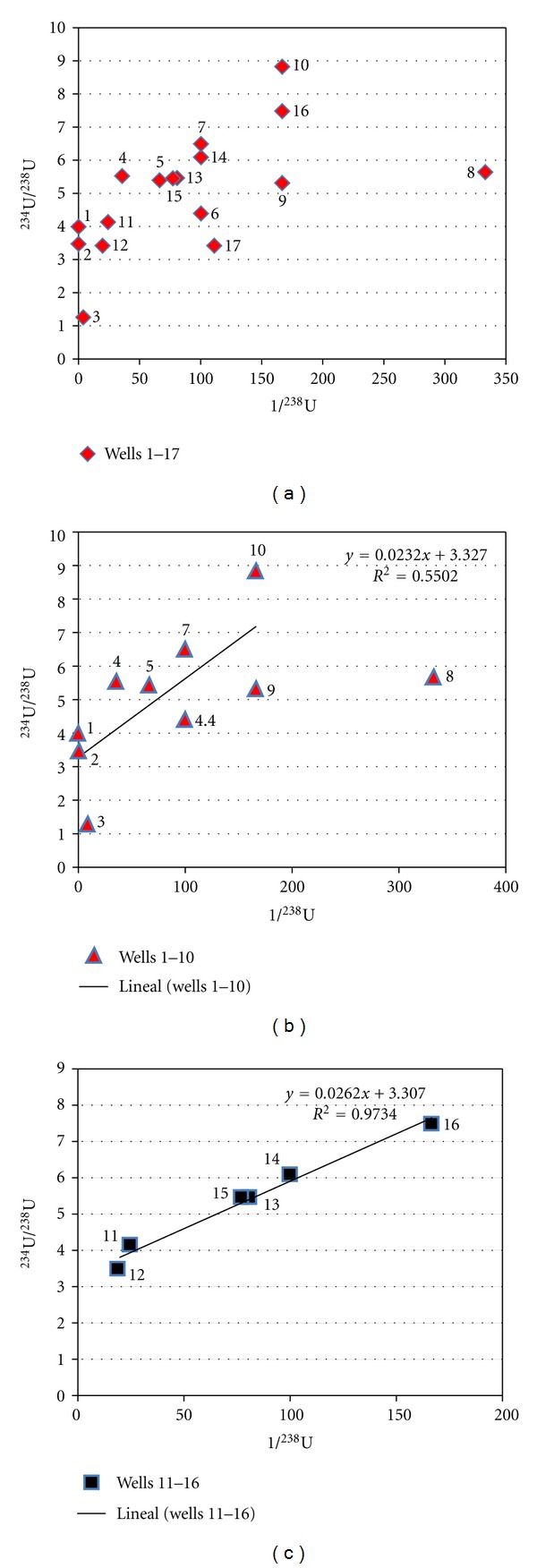
Correlations of ^234^U/^238^U versus 1/^238^U. Two sets of wells are considered, (a) total flux, uranium behavior in all sampled wells, (b) flux 1, uranium mobility in wells from 1 to 10, (c) flux 2, uranium mobility in wells from 11 to 16. Note: Wells 8 and 17 were not considered to fit in the linear correlations.

On the basis of knowing that ^234^U ions are more mobile than those of ^238^U, it has been documented that both the relative contribution of mixing systems and the interactions between aquifer substrate and water can be obtained by plotting ^234^U/^238^U (AR) versus 1/^238^U. It is possible to analyze the behavior of U-isotopes concentrations in each well and to establish any possible runoff way [35].  Figure 3 shows the correlation of ^234^U/^238^U activity ratio versus 1/^238^U (L/Bq) from this study. The direct experimental data plot as lineal function does not give any good information. 

**Figure 4:**
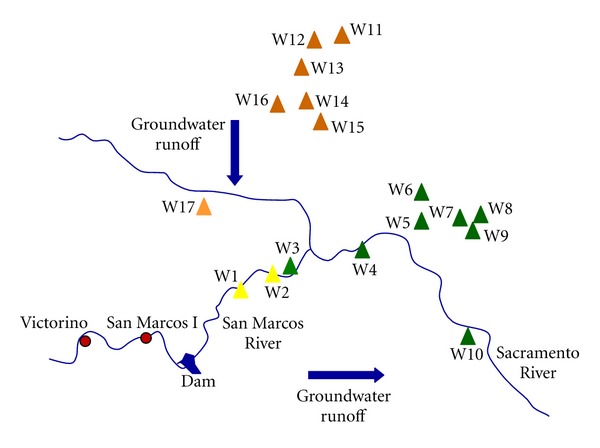
Direction of water runoff ways (represented by arrows) found in Sacramento valley, where flux 1 is localized at south and its direction is from southwest to northeast while flux 2 is localized at north and its direction is from north to south.

Sacramento valley is surrounded by three mountain ranges, and it is connected with Chihuahua valley by a “throat” at its southeast (see Figure 1).  Figure 3 results suggest that groundwater does not have only one runoff way. It is observed that a better Pearson correlation (*P*) was obtained if wells are presented in two territorial sets. The first set would correspond to flux 1 (Figure 3(b)), which is localized at the south of the valley, including wells from 1 to 10 (*P* = 0.74, *P* = 0.022); here the direction of flux 1 would be from southwest to southeast, as is showed in Figure 4. Second set (flux 2) is placed at north of Sacramento valley, including wells from 11 to 16. Flux 2 linear correlation has *P* = 0.99 (Figure 3(c)), where water runoff way would have north-south direction, see Figure 4. These groundwater fluxes have distinct U-isotopic characteristics (Figure 2(b)); they are indicating different dissolved uranium components. Following the interpretation described in [35], these components are as follows: (a) flux 1 (from SW to SE) shows a mixed linear pattern that ranged from a high uranium concentration and intermediate activity ratio to low uranium concentration and high activity ratio; (b) flux 2 (from N to S) is interpreted as a line of mixing between two points that indicate a pattern where the rock-water interaction is evolving. Flux 1 shows important changes related to uranium concentrations in every well sampled (see Figure 2). However, well 3 exhibits a drop of uranium concentration. This decrease of uranium concentration might be caused by dilution from mixing with water containing low uranium concentration. Thus, as shown in Figure 4 and following the water runoff of flux 2, this flux (which contains low uranium concentration and high activity ratio) will reach flux 1 at location between wells 2 and 3, getting uranium diluted water to well 3. After this point (well 3), water flux presents characteristics as low uranium concentrations and high activity ratio. Those uranium chemicals fractionation may be caused by different factors such as precipitation/coprecipitation, and adsorption, complexation [39]. Finally, wells 1 and 2 show high uranium contents in comparison with remaining wells. High uranium contents in water from wells 1 and 2 can be attributed to its proximity to San Marcos outcrops. 

### 3.2. Surface Water

Major ions contents were obtained using ICP-OES in water and suspended matter.  Table 3 shows concentrations of major ions, pH and TDS in water from San Marcos River, whereas Table 4 presents averages of major species content obtained for suspended matter from San Marcos River. 

Observed pH of water samples was close to neutral with average of 7.6, as is normally found in rivers flowing on igneous acid rocks. TDS concentrations vary along the path of river but generally display typical values of river waters with slow streams, ranging from 95 to 433 ppm. 

According to the results shown in Table 3, the river water might be classified as bicarbonated-calcic, showing linear correlation of Ca content with Mg content (*P* = 0.96, *P* = 0.000), HCO_3_ content (*P* = 0.79, *P* = 0.007), and TDS values (*P* = 0.89, *P* = 0.001). 

The radionuclides and other toxic elements in freshwater streams are strongly affected by suspended matter, so that in this investigation the elemental characterization of the suspended matter was also obtained. From results listed in Table 4, suspended matter is mainly composed by alkaline elements, some metals as well as sulfur; suspended mater displays Ca, Mg, and S concentrations higher than in water. 

To evaluate the uranium partitioning between water and suspended matter, the distribution coefficients (*K*_*d*_) are calculated, which are expressed as the activity concentration ratio between the particulate phase and the dissolved phase under equilibrium conditions [40]:

(1) $$k_{d}=\frac{\text{Activity}\;\text{concentration}\;\text{in}\;\text{suspended}\;\text{matter}}{\text{Activity}\;\text{concentration}\;\text{in}\;\text{water}}\frac{\left(\text{Bq}/\text{kg}\right)}{\left(\text{Bq}/\text{L}\right)}.$$

Activity concentrations of uranium in both water and suspended matter, collected in the river as well as their distribution coefficients (*K*_*d*_), are shown in Table 5. 

**Figure 5:**
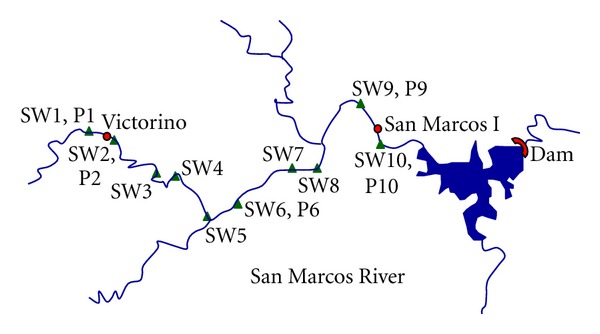
Points of both surface water and plant samples taken along to the San Marcos River. Note: the samples were taken in 2007.

The observed values of dissolved uranium in water samples, from 6 to 239 mBq/L, are not negligible. The distribution was not homogeneous. Here it is observed a sharp increase of the uranium concentration in water, in SW2 and SW3 points that are after Victorino outcrop. Also, it was observed that the uranium concentrations in water dropped when sampling points were farther from that outcrop, and a slightly increase after San Marcos I outcrop (see Figure 5). However, water from point SW5 shows uranium concentration as high as water from SW2 and SW3 points. Uranium concentration observed in this point can be attributed to groundwater input from a spring located just in that place. Likewise, this water sample has the highest concentrations of carbonates (see Table 3), which also contribute to have more uranium dissolved at observed pH. Published estimates for uranium activity concentration in filtered water fraction (size particles <0.45 *μ*m) in rivers range from 0.12 to 1200 mBq/L and have a global average of about 3.6 mBq/L [4]. In comparison with that, all the points sampled along of San Marcos River showed activities higher than this global average, where the highest uranium concentration in solution was found in the sampling point SW2 close to Victorino uranium outcrop. 

**Figure 6:**
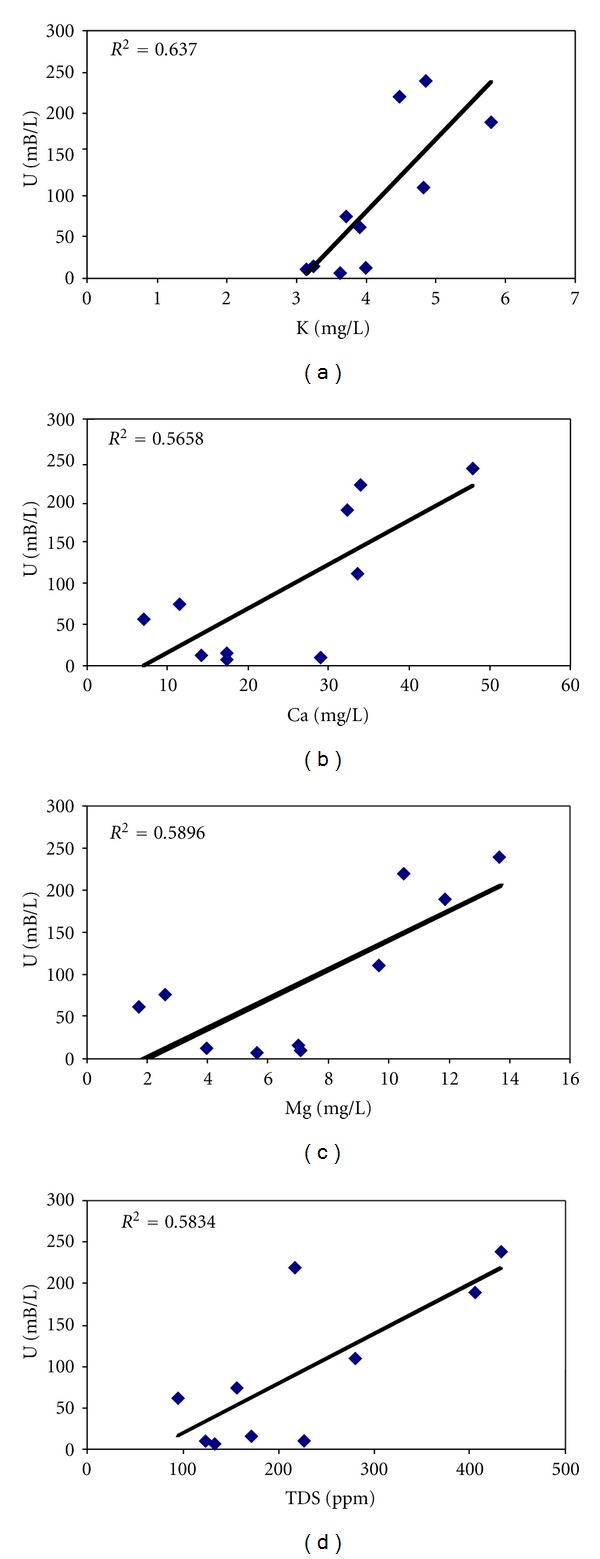
Correlations of specific activity of total U with the content of some elements in solution of water samples extracted from San Marcos River. (a) Correlation of U versus K, (b) correlation of U versus Ca, (c) correlation of U versus Mg, and (d) correlation of U versus TDS.

In surface and ground water, uranium tends to be complexed with carbonates at pH from 4 to 10, in dependence on the partial pressure *p*_co_2__ [5, 38]. However, in this study total uranium concentration showed a weak correlation with HCO_3_^−^ in solution.  Figure 6 shows the correlation between total dissolved uranium activity concentration with some major ions and TDS. Reyes-Cortes et al. [23] reported that in rocks from Victorino outcrop, uranium concentrations are in presence of iron and potassium contents. According to that, in these water samples the highest Pearson correlation was of uranium activity concentration with K concentration (*P* = 0.76, *P* < 0.011), followed by correlations with contents of Ca (*P* = 0.71, *P* = 0.022), Mg (*P* = 0.69, *P* = 0.028), and TDS (*P* = 0.66, *P* = 0.037). 

Taking into account the conditions of subsurface water runoff, the location pattern of the activity concentration of dissolved total uranium along the San Marcos River, and the correlation of dissolved total uranium to potassium and calcium, it may be concluded that uranium content in the different types of rocks forming the watershed and a contribution of groundwater with high uranium contents can explain the presence of uranium isotopes (mostly ^234^U) in surface water. These possibilities have been reported in previous published works, where the lithology of the bedrock is considered a key parameter for explaining a high uranium activity concentration [41]. 

The *K*_*d*_ values in suspended matter, reported in current literature, range from 10^0^ to 10^3^ (L/kg) [40]. The *K*_*d*_ results we have found in San Marcos River, see Table 5, ranged from 10^2^ to 10^5^, which means two order of magnitude higher than worldwide ranges. This fact might be due to the presence of high concentrations of both Fe and Ca-oxides that are present in this matrix. In these suspended matter samples, uranium shows a high correlation with both iron (*R* ^2^ = 0.85, *P* = 0.01) and calcium (*R* ^2^ = 0.85, *P* = 0.007). It is known that Fe-oxides or oxyhydroxides which might be present in water with pH ranged from 6.0 to 7.8 and that these iron oxides have a great uranium uptake capacity [42, 43]. This way, a great part of uranium that would be in solution is removed by Fe-oxides present in suspended matter. 

Activity concentrations of U-isotopes, measured by LSC, in water and suspended matter collected in the dam as well as their distribution coefficients (*K*_*d*_) are shown in Table 6. 

**Figure 7:**
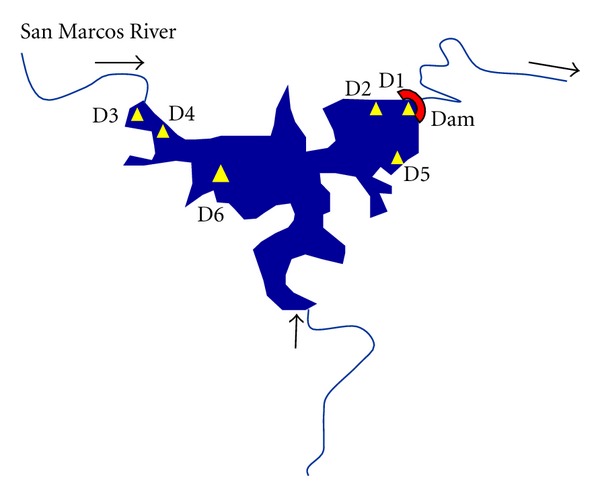
Water sample points in the San Marcos Dam. Note: water samples were taken in 2007.

Activity concentration of uranium in suspended matter from the dam was not as high as in the river (see Table 6). Here the water exhibits typical *K*_*d*_ values, only D3 and D4 points showed results of *K*_*d*_ around to maximum value reported in [40]. These results were expected because D3 and D4 points were sampled near to the river water input (see Figure 7). However, the dissolved uranium increases in the dam. There could be two causes for this behavior: firstly, due to dissolved uranium concentration found in water flowing from the San Marcos River, which is a source of recharge for the dam; Secondly, a spring in the dam which could contribute with high uranium concentration from subsurface water. Indeed, the highest uranium concentration in solution from the dam was found in the point where the spring is located (D2). 

### 3.3. Plants

Table 7 shows the results of activity concentration of total uranium obtained for plant samples, growing near to the outcrop and along of San Marcos river. Samples were divided in root, stem, and leaves. 

The values from Table 7 suggest that uranium trends to concentrate in leaf, following in roots and in steam. It has been reported greater activity concentration of uranium in leafy vegetables that in fruits and root vegetables [44, 45]. Authors explain that the activity in leaves is due to the contribution of several processes. These processes can be interception, absorption, resuspension, and translocation from roots to other components of the plant. 

In most cases the content of radionuclides is reported with positive linear correlation between soil and plant concentrations. However, the soil uranium concentration is not the only factor that should be taken into account when considering uranium uptake by plants. The transfer factor values can be influenced by causes such as soil characteristics, climatic conditions, type and age of plants, part of the plant concerned, physicochemical form of the radionuclides, and interfering elements [46, 47]. Other authors have found that the differences in the radionuclide translocations from roots to shoots are probably species-dependent [48, 49]. The roots are the part of plant that controls the absorption and transport to upper parts of many trace metals, including radionuclides. Some authors have found that the uranium concentrations in plants are affected by the same radioactive isotopes as the substrate, but in a nonlinear way [50]. In addition to that, it has been determined that the loss of transfer factor linearity is proper at low uranium concentrations in soil [47]. In the present study, the plant samples P2 and P9 are growing the closest to outcrops, Victorino and San Marcos I, respectively. Knowing that, and if we take in consideration only the uranium concentration in root, these samples (P2 and P9) do not show the highest uranium concentrations in plants. Only in sample 9 the high uranium concentration is found within root. However, the uranium concentration of whole plant (sum of root, stem, and leaves) is in concordance with the plants growing in soil with high uranium concentration, the nearest to uranium outcrops. 

Also, a sampling of the same plant species was carried out in July 2005, in the sampling point 5 (near to the San Marcos I outcrop); results are showed in Table 8. The plant was also divided in root, leaf, and stem, and the specific activity concentrations of ^238^U and ^234^U were determined by alpha spectrometry. In this analysis, it was obtained that the enhanced uranium concentration was in leaf and that uranium uptake by the plant is the same for ^238^U and ^234^U (AR is close to 1). It is interesting to notice that the results of the sampling in 2005 are lower than in the sampling of 2007. The decrement is approximately 80%. This may be related to age of plant samples. Anke et al. [51] have determined that uranium content decreases significantly when increasing the age of the vegetation. They found that the plants concentrate more uranium in their tissues in the seedling stage than in the flowery stage.* Baccharis salicifolia *is a plant that its flowery stage begins in July, so the first sampling (2005) was done when the plant was in flowering and the second sampling was carried out when the plants were young. Thus, we can attribute this difference of uranium concentrations to age of the plant. 

The obtained values demonstrate the possibility of uranium contamination in agriculture, although results are not categorically a reason for public health concern. 

### 3.4. Fish

In Table 9 are presented the ^238^U and ^234^U activity concentrations of clean muscle from fish, captured in San Marcos dam.

**Figure 8:**
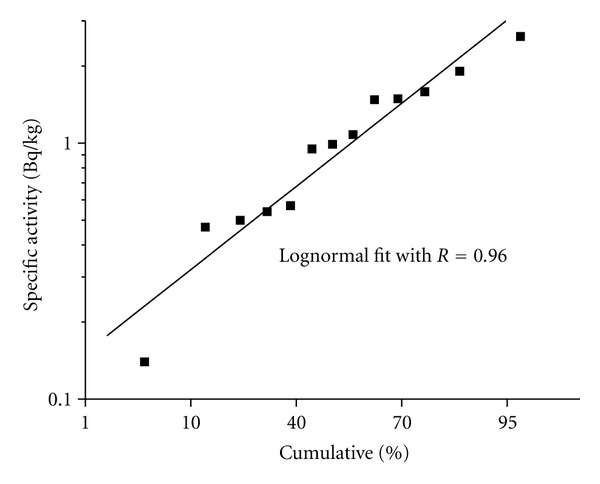
Lognormal probability pattern of specific activity concentrations of ^238^U in fish samples captured at San Marcos dam. Straight line represents the lineal fit of the experimental values. Note: fish samples were taken in 2005.

^234^U/^238^U activity ratio in most samples is about 2 or more. This result was expected, because uptake of uranium by fish can occur directly from water. The fish samples were taken from dam in 2005 sampling at the same time and place of water sampling. Water showed uranium activity concentration of 524 mBq/L (see above).  Figure 8 presents the probability graph of ^238^U activity concentrations, showing the lognormal character of the distribution. Out of 16 samples, two activity values were below the detection limit, and they were not used in the statistical calculations. Reported values by United Nations Scientific Committee on the Effects of Atomic Radiation [45] for the United States have an interval of activity concentration for ^238^U from 0.013 to 1.9 Bq/kg. In comparison with that, any of the statistical parameters of the lognormal distribution for ^238^U obtained in this study are much greater than the reference value of 0.03 Bq/kg reported in UNSCEAR 2000. This fact emphasizes the abnormal concentration of uranium in water and fish. 

The consumption rates of food and water, as well as the radionuclide concentrations are the main factors of ingestion dose of natural radionuclides. Although in Chihuahua State the consumption of fish is very low, we determined, to a hypothetical case, the effective dose (*E*_*D*_) of ^238^U and ^234^U that the local population could receive by ingestion of the fish in study. Here was taken into account the reference of annual intake of fish showed in UNSCEAR 2000 Report, where the consumption rate (kg/y) is of 5, 10, and 15 to infants, children, and adults, respectively. The results of effective dose are showed in Table 10. 

The effective dose reported in UNSCEAR 2000, only for ^234^U and ^238^U, is of 0.48, 0.54, and 0.53 *μ*Sv in infants, children, and adults, respectively. In comparison with that and under the hypothetical consumption rate described above of fish in study by the local population, it is observed (see Table 10) that the intake of uranium is up to 4 times greater than the reference value.

## 4. Conclusions

The activity concentrations of uranium obtained from groundwater samples show enhanced radioactivity in wells closest to the San Marcos region. Out of 17 sampled wells, only those three near to the outcrops have shown activity concentration in groundwater higher than Mexican health limits of alpha radioactivity. Using ^238^U activity concentration and ^234^U/^238^U activity ratio in ground water, a main flux into Sacramento valley running from the north, which is mixed with a second groundwater flux with high concentration of uranium leached from San Marcos zone can be inferred. 

The analyses of suspended matter and filtered water in the San Marcos River indicate that most of the uranium tends to be associated to the suspended matter and, in general, comparatively smaller fractions were present in the solution. In suspended matter, the uranium is associated mainly to Fe- and Ca-oxides; while uranium in solution can be correlated mainly to K and Ca, and in lesser extent to TDS. Highest dissolved uranium concentration in the river was found near to the Victorino outcrop. It was concluded that the dissolved uranium concentrations along the river depend primary on the lithology of the zone where the watershed is placed. The San Marcos dam shows high dissolved uranium concentrations due to two main sources, the contribution from the river and to the concentration from groundwater supply (spring). 

Observing the uranium concentrations obtained in the different parts of the plant, we may conclude that* Baccharis salicifolia* contain enhanced uranium in the leaves and take most uranium when the plant is young. In this plant species, the highest uranium concentrations were obtained in locations closest to uranium outcrops. This fact suggests that *Baccharis salicifolia* might considered a marker of uranium contamination in arid regions, where this plant is frequent. 

High uranium concentrations were found in fish. In a hypothetical case of this fish ingestion, the effective dose (^238^U and ^234^U) received by the local population would by far above the reference value given in the United Nations Scientific Committee on the Effects of Atomic Radiation 2000 Report. 

The highest natural radioactive isotope concentrations are to the northwest of the Chihuahua-Sacramento Valley, mainly in the mineralized deposits of San Marcos. The great values of activity ratio ^234^U/^238^U in ground and some surface water, not only near the outcrops, indicate that the San Marcos region is almost totality an uraniferous zone. By all these considerations, it is concluded that in this zone there is natural contamination by uranium in all environmental samples analyzed in the present work.

## Figures and Tables

**Table 1:** Sampling stations and physicochemical parameters of the groundwater. Note: W1 and W2 were collected in 2005 (rainy season) and the rest of the samples in 2007 (dry season).

Well site	pH	TDS (ppm)	*T *(°C)	Depth (m)	North	West	Elevation (m)
1	6.8	80	20	15	28° 49′ 9.8′′	106° 19′ 5′′	1623
2	6.8	120	20	10	28° 49′ 38.6′′	106° 17′ 53′′	1603
3	7.1	190	20	240	28° 49^'^ 52.4′′	106° 17^'^ 17′′	1596
4	7.4	160	20	160	28° 51^'^ 7.1′′	106° 14^'^ 36′′	1576
5	7.4	180	22	45	28° 51^'^ 30′′	106° 12^'^ 20′′	1556
6	7.6	240	22	s.w.*	28° 52^'^ 21′′	106° 12^'^ 39′′	1563
7	7.7	180	22	s.w.	28° 51^'^ 29′′	106° 10^'^ 59′′	1539
8	7.4	190	20	100	28° 52^'^ 18′′	106° 10^'^ 35′′	1530
9	7.7	210	22	100	28° 51^'^ 40′′	106° 10^'^ 18′′	1533
10	7.9	250	20	120	28° 50^'^ 67′′	106° 11^'^ 48′′	1537
11	7.4	210	20	90	28° 56^'^ 93′′	106° 15^'^ 65′′	1592
12	7.5	310	20	150	28° 56^'^ 74′′	106° 16^'^ 62′′	1607
13	7.5	290	22	180	28° 56^'^ 93′′	106° 18^'^ 41′′	1630
14	7.6	170	22	200	28° 54^'^ 83′′	106° 16^'^ 61′′	1610
15	7.6	180	22	s.w.	28° 54^'^ 83′′	106° 16^'^ 14′′	1607
16	7.4	170	22	150	28° 54^'^ 83′′	106° 17^'^ 6′′	1630
17	6.8	150	20	s.w.	28° 51^'^ 51′′	106° 19^'^ 72′′	1641

* s.w: shallow wells.

**Table 2:** Activity concentrations of ^238^U and ^234^U isotopes and their uncertainty, as well as ^234^U/^238^U activity ratio, in groundwater samples extracted from wells of Sacramento valley.

Well	^238^U (mBq/L)	^234^U (mBq/L)	^234^U/^238^U	Sampling year
1	6750 ± 450	27030 ± 1040	4 ± 0.3	2005
2	6070 ± 400	21200 ± 840	3.5 ± 0.3	2005
3	250 ± 1.0	311 ± 1.2	1.3 ± 0.01	2007
4	28 ± 0.7	155 ± 1.6	5.5 ± 0.1	2007
5	15 ± 0.7	82 ± 1.1	5.4 ± 0.3	2007
6	10 ± 0.4	44 ± 0.6	4.4 ± 0.2	2007
7	10 ± 0.4	65 ± 1.0	6.5 ± 0.3	2007
8	3 ± 0.3	17 ± 0.6	5.7 ± 0.6	2007
9	6 ± 0.4	32 ± 0.7	5.3 ± 0.4	2007
10	6 ± 0.4	53 ± 0.9	8.8 ± 0.6	2007
11	41 ± 0.8	170 ± 1.6	4.1 ± 0.1	2007
12	51 ± 0.9	175 ± 1.6	3.4 ± 0.1	2007
13	12 ± 0.5	68 ± 0.9	5.5 ± 0.2	2007
14	10 ± 0.4	61 ± 1.0	6.1 ± 0.3	2007
15	13 ± 0.5	71 ± 1.0	5.5 ± 0.2	2007
16	6 ± 0.4	45 ± 0.8	7.5 ± 0.5	2007
17	9 ± 0.4	31 ± 0.7	3.4 ± 0.2	2007

**Table 3:** Sampling parameters and major ions concentrations (mg_ion_/L or ppm) dissolved in water from San Marcos River. Samples were collected in 2007.

Sample	pH	TDS	HCO_3_^−^	SO_4_^−2^	NO_3_^−^	Cl^−^	Na^+^	Ca^+2^	Mg^+2^	K^+^
SW1	7.6	227	135	57	1	10	5	29	7	3
SW2	7.6	433	248	91	2	10	33	48	14	5
SW3	6.9	217	207	22	2	33	12	34	11	5
SW4	7.9	172	198	1	2	1	17	17	7	3
SW5	7.3	405	303	5	9	0.5	25	32	12	6
SW6	8.1	280	233	3	2	2	24	34	10	5
SW7	7.5	133	168	2	2	0.2	17	17	6	4
SW8	7.9	123	129	1	1	2	13	14	4	4
SW9	7.5	95	56	0.1	2	38	8	7	2	4
SW10	7.4	157	73	13	1	38	5	12	3	4

**Table 4:** Concentrations of some elements (mg_ion_/kg or ppm) detected in suspended matter extracted from water at San Marcos River.

Element	Minimum value	Maximum value	Average
Ca	1127	6710	2822
Fe	85	418	223
Mg	124	831	333
Mn	2	10.4	3.8
Pb	7.3	26	14.5
Zn	18.7	297.3	139.5
S	432.4	2559	1142.3
K	102.6	799	398.2
Na	444	2619	1485

**Table 5:** Activity concentrations of total uranium and their uncertainty, as well as *K*_*d*_ coefficient, in water (U_w_) and in suspended matter (U_sm_), at San Marcos River. Samples were collected in 2007.

Sample	U_w_ (mBq/L)	U_sm_ (kBq/kg)	K_d_ (L/kg)	Comments
SW1	10 ± 0.4	1.3 ± 0.1	1.3 × 10^5^	Before Victorino
SW2	239 ± 18	0.32 ± 0.003	1.3 × 10^3^	After Victorino
SW3	220 ± 23	0.21 ± 0.02	9.6 × 10^2^	
SW4	15 ± 0.5	0.66 ± 0.06	4.4 × 10^4^	
SW5	189 ± 15	0.20 ± 0.02	1.1 × 10^3^	Confluence
SW6	110 ± 1	n.d.*	—	
SW7	6 ± 0.4	0.03 ± 0.002	5.0 × 10^3^	
SW8	12 ± 0.4	0.17 ± 0.01	1.4 × 10^4^	
SW9	61 ± 7	0.022 ± 0.002	3.6 × 10^2^	Before San Marcos I
SW10	74 ± 14	n.d.	—	After San Marcos I

*n.d. means below detection limit.

**Table 6:** Activity concentrations and their uncertainties of total uranium, as well as *K*_*d*_ coefficients, in solution (U_d_) and in suspended matter (U_sm_), from water samples extracted at San Marcos dam. Samples were collected in 2007.

Sample	U_d_ (mBq/L)	U_sm_ (kBq/kg)	*K*_d_ (L/kg)	Comments
D1	197 ± 18	0.15 ± 0.01	7.6 × 10^2^	Dam barrier
D2	524 ± 48	0.16 ± 0.01	3.1 × 10^2^	Groundwater input (spring)
D3	210 ± 18	0.22 ± 0.02	1.0 × 10^3^	
D4	215 ± 20	0.20 ± 0.02	9.3 × 10^2^	River water input
D5	289 ± 25	0.11 ± 0.01	3.8 × 10^2^	
D6	143 ± 14	n.d.*	—	

* n.d. means below detection limit.

**Table 7:** Specific activity concentrations of total uranium and their uncertainty in plant samples taken along to the San Marcos River. Sampling was performed in March-April of 2007.

Sample	U_t_ root (Bq/kg)	U_t_ stem (Bq/kg)	U_t_ leaf (Bq/kg)
P1	17.2 ± 1.3	22.9 ± 1.9	25.1 ± 1.9
P2	15.7 ± 1.2	20.3 ± 1.5	24.9 ± 1.8
P6	20.5 ± 1.5	11.1 ± 0.9	25.8 ± 2.0
P9	22.6 ± 1.8	13.9 ± 1.0	13.5 ± 1.0
P10	19.1 ± 1.5	35.2 ± 3.1	36.7 ± 3.2

**Table 8:** Activity concentrations, their uncertainties of uranium, and corresponding AR (^234^U/^238^U) in the plant sample taken near to the San Marcos River (point 5). Sampling was carried out in August of 2005.

Plant	^238^U (Bq/kg)	^234^U (Bq/Kg)	^234^U/^238^U
Root	1.7 ± 0.1	1.9 ± 0.1	1.1 ± 0.09
leaf	5.3 ± 0.1	5.8 ± 0.1	1.1 ± 0.03
Stem	1.1 ± 0.03	1.1 ± 0.03	1 ± 0.04

**Table 9:** Activity concentrations of ^238^U and ^234^U isotopes and their uncertainties (in parenthesis) and AR (^234^U/^238^U) for fish samples from San Marcos Dam. Samples were collected in 2005.

Sample	^238^U (Bq/kg)	^234^U (Bq/Kg)	^234^U/^238^U
F1	1.0 ± 0.2	2.0 ± 0.3	2.1 ± 0.5
F2	1.6 ± 0.2	2.6 ± 0.2	1.6 ± 0.2
F3	1.9 ± 0.3	1.7 ± 0.3	0.9 ± 0.2
F4	1.5 ± 0.1	2.7 ± 0.1	1.8 ± 0.1
F5	1.1 ± 0.1	1.8 ± 0.1	1.7 ± 0.2
F6	2.6 ± 0.2	4.5 ± 0.2	1.7 ± 0.1
F8	0.5 ± 0.1	1.4 ± 0.1	2.9 ± 0.6
F9	0.6 ± 0.1	1.2 ± 0.1	2.1 ± 0.4
F10	0.5 ± 0.1	1.6 ± 0.1	3.5 ± 0.7
F12	0.14 ± 0.06	0.6 ± 0.1	4.5 ± 2.1
F13	1.5 ± 0.1	2.0 ± 0.1	1.3 ± 0.1
F15	1.0 ± 0.1	2.1 ± 0.2	2.1 ± 0.3
F16	0.5 ± 0.1	2.0 ± 0.3	3.7 ± 0.9

**Table 10:** Effective dose (*E*_*d*_) of ^238^U and ^234^U calculated for a hypothetical case.

Radionuclide	Effective dose coefficient (*μ*Sv/Bq)*			*E*_*D*_ (*μ*Sv y^−1^)		
	Infants	Children	Adults	Infants	Children	Adults
^238^U	0.12	0.068	0.045	0.67	0.76	0.75
^234^U	0.13	0.074	0.049	1.31	1.49	1.48

Total				1.98	2.25	2.23

*Values taken from UNSCEAR 2000 Report.
